# An investigation of the contribution of different turn speeds during standing turns in individuals with and without Parkinson’s disease

**DOI:** 10.1038/s41598-022-27217-4

**Published:** 2022-12-29

**Authors:** Fuengfa Khobkhun, Paulo Roberto Pereira Santiago, Ariany Klein Tahara, Prachaya Srivanitchapoom, Jim Richards

**Affiliations:** 1 Parkinson Movement and Research Collaboration Laboratory, Faculty of Physical Therapy, Mahidol University, Salaya, 73170 Nakhon Pathom Thailand; 2 Biomechanics and Motor Control Laboratory, School of Physical Education and Sport of Ribeirão Preto, University of São Paulo, Ribeirão Preto, Brazil; 3 Program in Rehabilitation and Functional Performance, Ribeirão Preto Medical School, University of São Paulo, Ribeirão Preto, Brazil; 4 Division of Neurology, Department of Medicine, Faculty of Medicine, Siriraj Hospital, Mahidol University, Bangkok, Thailand; 5 Allied Health Research Unit, School of Sport and Health Sciences, University of Central Lancashire, Preston, UK

**Keywords:** Diseases, Health care, Neurology, Risk factors, Signs and symptoms

## Abstract

Issues around turning can impair daily tasks and trigger episodes of freezing of gait in individuals with Parkinson's disease (PD). Slow speeds associated with aging produce a more en-bloc movement strategy which have been linked with falls while turning. However, the influence of speed of turning on the complex whole-body coordination considering eye movements, turning kinematics, and stepping characteristics during turning has not been examined. The aim of this study was to investigate if individuals with PD have a different response to changes in turning speed compared to healthy older adults during 180° standing turns. 20 individuals with PD and 20 healthy age matched adults participated in this study. Data were collected during clockwise and counter-clockwise turns at three self-selected speeds in a randomised order: (a) normal; (b) faster than normal; and (c) slower than normal. Eye movement and turning kinematics were investigated using electrooculography and Inertial Measurement Units. Mixed Model Analysis of Variance (MM ANOVA) tests with post hoc pairwise comparisons were performed to assess the differences between groups and turning speed. In addition, further post hoc Repeated Measures ANOVA (RM ANOVA) tests were performed if any significant interactions were seen between groups and turning speed. Significant interaction effects were found in eye movement and turning kinematics, and the RM ANOVA showed significant main effects for turning speeds within the PD and the control groups. Turning slowly resulted in similar alterations in eye movement, turning kinematics and stepping characteristics in the PD group and the healthy controls. However, individuals with PD showed a different response to the healthy controls, with a greater delay in eye movement and onset latency of segments in turning kinematics and step variables between the different speeds. These findings help our understanding regarding the turning strategies in individuals with PD. The incorporation of guidance with regard to faster turning speeds may be useful in the management of individuals with PD. Clinical training using different turn directions and speeds may improve coordination, increase confidence and reduce the risk of falling.

## Introduction

Parkinson’s disease (PD) is the second most common neurodegenerative disease caused by the loss of predominantly dopaminergic neurons due to the degeneration of substantia nigra pars compacta (SNpc) which leads to functional changes in the nucleus of the basal ganglia and in the nucleus of the brainstem, which can be associated with the presentation of movement disorders^1^. The cardinal features of individuals with PD include symptoms of bradykinesia, rigidity, resting tremor and postural instability, and also a variety of other motor and non-motor symptoms^2^. Motor symptoms can include impairments in; balance, postural instability, flexibility, strength, trunk stability, segment coordination and freezing, all of which can lead to difficulties with turning and deterioration in the quality of movement in individuals with PD^3–5^. Postural instability and balance impairments are common manifestations found in the middle and severe stages within the progression of PD^6^ which is believed to result from postural reflex dysfunction caused by neck and trunk rigidity, bradykinesia (slow movements), sensory impairments (such as deficits in vision), vestibular function and proprioception as well as the shuffling gait characterised by abnormally small and frequent steps^7–11^. Several previous studies have documented that postural instability contributes to the difficulties in turning that emerge during the disease progression and advanced severity of PD, specifically between 2.5 and 4 on the modified Hoehn and Yahr staging scale^5,7,10^.

Before changing direction, healthy adults usually move their gaze in the direction of the turn, this behaviour being believed to represent an important part of a top-down coordination sequence^12–14^. The timing and nature of eye movements, as well as the relative rotation between body segments observed during turning, are heavily influenced by the speed and size of the rotation^9,15^. Turning difficulties in individuals with PD are commonly characterized by an increase in turn duration, the number of steps taken to turn and an en-bloc strategy which is defined as the disrupted time and coordination of axial segment movements^3,16–18^. It has been suggested that eye movement problems in individuals with PD may contribute towards deficits of whole-body coordination during turning^12^. In addition, turning speed has been reported to be an important factor associated with turning difficulties in individuals in the early to middle stages of PD^19^. Turns are often considered as a difficult locomotor activity by older adults^20^ and frequently lead to falls in individuals with PD^3,12,21^. About 70% of individuals with PD report activity limitations due to problems during turning, which has been shown to lead to social isolation^2,3^. Furthermore, falls in individuals with PD during 180 degree turns have been associated with activities such as negotiating check outs at the supermarket^9^, or navigating around an obstacle^22^, which can lead to injuries and loss of independence^16^. However, the relationship between turning dysfunction in individuals with PD and falling is complex, and the factors that underlie turning problems in PD have not been fully determined. More basic research is needed to fully understand how standing turns are coordinated and the consequences of changing the speed of the turns in individuals with PD. To date, turning measurements have previously been achieved by using Inertial Measurement Units (IMUs), using signal characteristics such as angular displacement and velocity^10,14,23–25^. It has been demonstrated that IMUs could be used in isolation to gather relevant data from individuals with PD, and potentially allowing continuous monitoring of mobility outside of the clinical and laboratory environments.

Therefore, the aim of this study was to investigate turning characteristics with different turning speeds in individuals with PD in comparison to aged matched healthy older adults during standing turns. We hypothesized that changing turn speed would result in changes in turning characteristics in individuals with PD but not in healthy older adults. This present study may help clinicians and could provide information to inform rehabilitation strategies and the development of specific turning programmes to reduce the fall risks related to turning tasks in individuals with PD.

## Methods

### Study design and participants

G*Power statistical software was used to determine the sample size required using the head onset latency based on a previous study that used a similar methodology^12^. A sample size of 12 participants per group was determined to be sufficient based on a statistical power of 90% at a significance level of 5% to detect any outcome differences between groups. However, a previous study in healthy adults using this protocol^14^ showed 15 participants to be sufficient for within group analysis, therefore the sample size was set to at least 20 participants per group, an older adult group and a group of individuals with PD, to allow for any dropouts or missing data.

This study was conducted in the Parkinson Movement and Research Collaboration laboratory (PMARC lab), Faculty of Physical Therapy at Mahidol University, Thailand. The inclusion criteria for the PD group were: individuals with PD who had been clinically diagnosed with idiopathic PD stages 2.5–3 assessed using the modified Hoehn and Yahr scale by a neurologist, aged between 50 and 75 years, stable without any changes in anti-PD medication for at least one month prior to participation in the study. This ensured that as many confounding effects on the turning performance as possible were minimized, particularly as regards variations in dosage of antiparkinsonian drugs during testing, able to walk without the need for any kind of assistance and be able to follow commands and instructions and show no signs of cognitive impairment which was assessed using the mini-Thai mental state examination with a score of ≥ 24/30^26^. The exclusion criteria were: clinically diagnosed with dementia or other neurological or cardiopulmonary diseases, musculoskeletal problems that could influence the test performance such as arthritis or severe leg pain, and visual problems that could not be adjusted with lenses or glasses. The control group were healthy older adults who were matched by age and gender to the PD group. Exclusion criteria were: self-reporting of any neurological or musculoskeletal problems, cognitive impairment assessed using the mini-Thai mental state examination with a score of ≥ 24/30^26^, use of medication for anxiety and/or dizziness, the use of an assistive device for walking. The study was approved by the Ethical Committee of Mahidol University Institutional Review Board, Mahidol University, Thailand (COA MU-CIRB 2020/040.1803) and complied with the standard guideline of the Declaration of Helsinki. All participants signed an informed consent form before data collection. In addition, informed consent was obtained from all participants involved in in the study and all participants gave the permission for the publication.

### Turning difficulty questionnaires and clinical assessments

Questionnaires addressing turning difficulty^20^ were recorded. In addition, the total motor score of the Movement Disorders Society-Unified Parkinson’s Disease Rating Scale (MDS-UPDRS) was used to evaluate symptoms in the PD group.

### Eye movement, turning kinematics and stepping characteristic assessments

Eye movement, turning kinematics and stepping characteristics were assessed using a Bluegain wireless electrooculography system (EOG) and Inertial Measurement Units (IMU) sensors, respectively. The presentation of visual cues and a simultaneously marked time point within the EOG data acquisition software and the synchronisation of XSENS data streams were controlled using a LabVIEW programme^14,23,27^.

#### Eye movement assessment

The BlueGain wireless electrooculography system (EOG) (Cambridge Research System Ltd., UK) was used to measure eye movement variables in terms of fast phase characteristics at a sampling frequency of 1000 Hz, similar to previous published methodologies^15,27^. Two disposable surface electrodes were placed on the outer canthi of the eyes and a reference electrode was placed on the centre of the forehead. A dual low-pass fourth-order Butterworth filter using a cut-off frequency of 30 Hz were applied to EOG dataset prior to calculating fast phase characteristics.

#### Turning kinematics and stepping characteristic assessments

**Figure 1:**
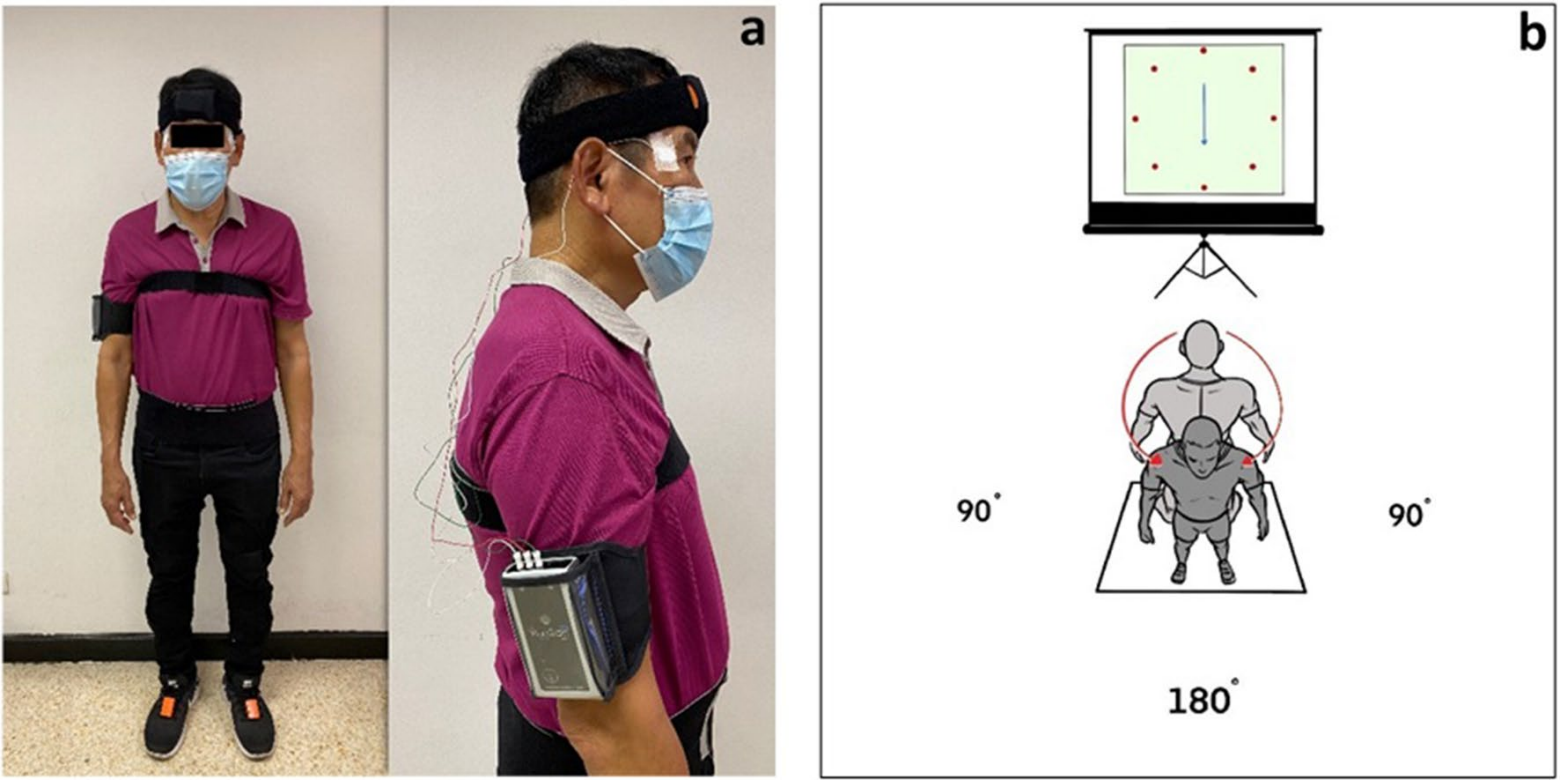
(**a**) Five inertial measurement units and electrooculography attachment, and (**b**) experimental setting protocol.

IMU sensors using XSENS Motion Capture (Xsens Technologies, the Netherlands), were used to measure turning characteristics in terms of turning kinematics and stepping characteristics at a sampling frequency of 100 Hz while participants performed a turn on level ground through 180° in a standing position. Five sensors were attached to the centre of the forehead, middle thorax, pelvis and the centre of the left and right foot using Velcro straps (Fig. 1)^14,27^. A dual low-pass fourth-order Butterworth filter using a cut-off frequency of 6 Hz were applied to IMU data prior to calculating turning kinematics and stepping characteristics. The specific parameters for the turning kinematics as markers of axial segment coordination included: reorientation onset time (seconds (s)) of eye, head, thorax, pelvis and feet, peak head yaw velocity (degrees/second (°s^−1^)) and peak head-segment angular separation angle (degrees (°)). In order to produce velocity and acceleration profiles for each segment, the displacement profiles were first differentiated. The earliest time point prior to a segment displacement of 5° with a velocity > 0° s^−1^ was used to calculate the rotation onset for each segment. The first zero crossing in the velocity profile after the completion of the segment rotation was used to define the end of rotation. The onset and offset latencies from the axial segments were used to produce time-normalized profiles for the axial segments since the time course of the turn trials had a variable duration. The data were normalized to the time points between the onset of the head yaw and the final axial offset in MATLAB, and the angular separation profiles for the head-thorax, head-pelvis, and thorax-pelvis were created by subtracting one profile from another.

The stepping characteristics recorded included; step count number (number (N)), step duration (seconds (s)), step size (degrees (°)) and step frequency (steps/seconds (Hz)). The temporal characteristics of individuals' steps, including step onset time and step duration (seconds), were calculated using step onset and step placement times during the turn. The yaw rotation of the foot (degrees) during the swing phase of each step while turning was used to calculate the average step size. The number of steps taken divided by the time of steps duration was used to calculate the step frequency.

All data processing was analysed using a previously validated program for the assessment of axial segment coordination during turning^14,15,23,27^.

### Turning protocol for data collection and data processing

On the day of the assessment, all PD participants were asked to take their medication less than 30 min before testing. All participants were asked to stand approximately 1 m in front of a projector screen. A practice phase was conducted prior to the experimental session to limit turn speed variability within and between participants. The participant began the practice phase by viewing the turn demonstration videos used in the experimental trials, then started to turn in response to audio signals and turn to the direction shown on the projector screen. The participant was instructed to start turning after the signal finished and complete a 180 degree turn. When instructed to "turn around" and point in a new direction, all participants invariably ended up with their heads, bodies, and feet aligned with the new direction of travel. None of the subjects gave any appearance of confusion at any point about how to align their body segments. These practice trials continued until both the investigator and participant were satisfied with the level of confidence in performing the turns. Reorientation of body segments were guided by the direction of an animated clock as was the speed of the turn. Data were collected in a randomised order for three turn conditions: a self-selected natural turn speed (normal), a self-selected faster than normal speed (faster) and finally at a self-selected slower than normal speed (slower), which were performed in randomly selected clockwise or counter-clockwise directions. Trials were recorded for turns to both the left and right sides, with a total of 6 trials being completed. All dependent variables were processed, analysed and extracted from MATLAB (R2022a) using a previously validated script and methodology^14,15,23,27^. Finally, all participants were asked to rate the easiest and most difficult turning speed.

### Statistical analysis

The data distribution was tested using the Shapiro–Wilk test and all variables were found to be normally distributed and suitable for parametric testing. Therefore, as the factors involved two groups (healthy control or PD), two directions (left or right), and three speeds (faster, normal or slower) a Mixed Model Analysis of Variance (MM ANOVA) with post hoc pairwise comparisons were performed on turning characteristics and eye movement variables. There were no effects of direction on any measures, therefore, data was collapsed resulting in a 2 × 3 MM ANOVA. In addition, further post hoc testing using Repeated Measures ANOVA (RM ANOVA) tests were performed if any significant interactions were seen from the MM ANOVA. Statistical significance level was set at *p* < 0.05, and all statistical analysis was performed using SPSS version 24.0 (IBM Corporation, Armonk, NY).

### Ethics approval and consent to participate

The study was approved by the Ethical Committee of Mahidol University Institutional Review Board, Mahidol University, Thailand (COA MU-CIRB 2020/040.1803) and complied with the standard guideline of the Declaration of Helsinki. All participants signed an informed consent form before data collection. In addition, informed consent was obtained from all participants involved in in the study and all participants gave the permission for the publication.

## Results

Forty-eight individuals (24 from the PD group and 24 from the control group) were recruited to this study. However, eight individuals (four from the PD group and four from the control group) did not meet the criteria. Therefore, 40 participants in total (20 participants for each group) were included in the analysis. The demographic data of healthy older adults (control) and PD groups are shown in Table 1. There were no significant differences between groups with regard to age, body mass index, cognitive ability and underlying disease which was tested using independent t-tests (*p* > 0.05), with only turning difficulty showing significant differences between groups (*p* < 0.05) with 60% of the PD group reporting difficulties versus 10% in the healthy group.

**Table 1:** Demographic data for the individuals with Parkinson’s disease (PD) (n = 20) and the healthy older adult group (n = 20).

Demographic	PD group (n = 20)	Control group (n = 20)	*p*-value
Age (years ± SD)	66.50 ± 4.17	67.00 ± 4.37	0.797
Body mass index (kg/m^2^ ± SD)	23.21 ± 2.91	23.39 ± 4.85	0.921
Gender, male/female (n)	10/10	10/10	1
Mini-Mental State Examination (scores ± SD)	29.75 ± 4.72	29.90 ± 5.14	0.998
**Underlying disease (n, %)**			
Hypertension	5 (50)	4 (40)	0.645
Diabetes mellitus	3 (30)	2 (20)	0.587
Others	2 (20)	4 (40)	0.434
Onset duration of Parkinson’s disease (years ± SD)	6.36 ± 3.51	–	–
Modified Hoehn and Yahr scale (stages (n))	2.5 (8)	–	–
	3 (12)		
MDS-UPDRS part III motor examination (on-phase, score ± SD)	29.64 ± 4.63	–	–
Taking l-DOPA (n, %)	18 (90)		
With others (n, %)	14 (72)	–	–
Alone (n, %)	4 (28)	–	–
**Reported turning difficulty, (n, %)**			
Yes	12 (60)	2 (10)	0.012*
No	8 (40)	18 (90)	0.035*

*MDS-UPDRS* Movement Disorder Society Unified Parkinson’s Disease Rating Scale, *SD* standard deviation.

*Significant difference (*p* < 0.05) by independent t-test.

For the eye movement variables, no interactions or main effects were seen in the case of the nystagmus fast phase frequency eye movement characteristics, (Table 2). However, a significant interaction (*p* < 0.05) was found in the first fast phase amplitude and velocity, maximum fast phase amplitude and peak fast phase velocity eye movements. In addition, there was a main effect of turn speed in the number of fast phase eye movements. A further analysis using a RM ANOVA revealed a main effect of turning speed (*p* < 0.05) and post-hoc pairwise comparisons showed that first and peak fast phase amplitude and velocity, and number of fast phase eye movements increased with an increase in turn speed in both groups (Table 3).

**Table 2:** The interaction between healthy age matched control and Parkinson’s disease (PD) groups and different speeds for turning characteristics variables shown as mean and standard deviations (SD), performed by mixed model analysis of variance.

Variables	PD group (n = 20)			Control group (n = 20)			Main effects	
	Faster	Normal	Slower	Faster	Normal	Slower	Group effect	Speed effect
							*p*-value (η_p_^2^)	*p*-value (η_p_^2^)
**Eye movement variables**								
1st fast phase amplitude (º)^+^	34.7 ± 7.7	29.1 ± 8.4	26.1 ± 0.1	30.5 ± 7.8	32.7 ± 8.7	30.8 ± 10.2	0.389 (0.007)	0.113 (0.05)
1st fast phase velocity (°s^–1^)^+^	372.1 ± 65.6	336.9 ± 76.0	321.2 ± 62.7	334.5 ± 51.6	286.8 ± 64.1	280.9 ± 64.1	0.011* (0.08)	0.382 (0.03)
Maximum fast phase amplitude (º)^+^	34.2 ± 6.9	37.9 ± 6.3	37.5 ± 6.9	38.4 ± 8.4	33.6 ± 6.8	32.6 ± 5.9	0.192 (0.02)	0.731 (0.001)
Peak fast phase velocity (°s^–1^)^+^	492.7 ± 68.9	447.1 ± 81.1	373.8 ± 62.3	437.0 ± 89.5	389.9 ± 60.6	379.3 ± 60.6	0.042* (0.05)	0.310 (0.23)
Number of fast phase (n)	3.1 ± 0.9	5.0 ± 1.2	6.3 ± 1.6	3.2 ± 0.8	4.6 ± 1.3	6.3 ± 1.9	0.750 (0.001)	0.011* (0.63)
Nystagmus fast phase frequency (n)	2.1 ± 0.6	2.4 ± 0.64	2.54 ± 0.64	2.3 ± 0.6	2.3 ± 0.6	2.4 ± 0.7	0.756 (< 0.001)	0.272 (0.03)
**Turning kinematics variables**								
***Segment reorientation onset latencies***								
Eye (s)	0.56 ± 0.07	0.61 ± 0.08	0.64 ± 0.11	0.53 ± 0.07	0.59 ± 0.07	0.63 ± 0.11	0.406 (0.006)	< 0.001* (0.17)
Head (s)	0.56 ± 0.08	0.63 ± 0.05	0.70 ± 0.05	0.54 ± 0.06	0.62 ± 0.07	0.66 ± 0.69	0.039* (0.50)	< 0.001* (0.53)
Thorax (s)	0.56 ± 0.08	0.63 ± 0.05	0.70 ± 0.05	0.54 ± 0.06	0.62 ± 0.07	0.67 ± 0.07	0.062 (0.96)	< 0.001* (0.37)
Pelvis (s)	0.54 ± 0.07	0.60 ± 0.05	0.68 ± 0.05	0.52 ± 0.05	0.60 ± 0.08	0.64 ± 0.08	0.135 (0.02)	< 0.001* (0.52)
Leading foot (s)^+^	0.71 ± 0.08	0.81 ± 0.06	0.86 ± 0.10	0.64 ± 0.09	0.75 ± 0.11	0.85 ± 0.09	0.795 (< 0.001)	< 0.001* (0.51)
Trailing foot (s)^+^	0.95 ± 0.14	1.08 ± 0.09	1.13 ± 0.13	0.88 ± 0.12	0.99 ± 0.14	1.11 ± 0.11	0.501 (0.004)	< 0.001* (0.47)
Peak head yaw velocity (°s^−1^)	251.0 ± 37.1	175.6 ± 22.6	130.5 ± 17.7	283.6 ± 57.8	186.3 ± 24.2	133.9 ± 24.9	0.013* (0.08)	< 0.001* (0.83)
Peak head-thorax angular separation (°)	18.6 ± 7.2	12.7 ± 7.4	9.7 ± 5.3	21.5 ± 9.9	15.4 ± 7.3	14.8 ± 5.2	0.008* (0.07)	< 0.001* (0.26)
Peak head-pelvis angular separation (°)	25.5 ± 2.8	21.6 ± 1.6	19.9 ± 2.2	30.2 ± 2.8	25.2 ± 5.8	21.7 ± 5.8	< 0.001* (0.20)	0.021* (0.10)
**Stepping variables**								
Number of steps (n)	3.62 ± 0.34	4.26 ± 0.43	4.91 ± 0.76	3.26 ± 0.43	3.55 ± 0.22	4.24 ± 0.90	< 0.001* (0.32)	< 0.001* (0.49)
Step frequency (Hz)	3.22 ± 0.36	3.67 ± 0.35	4.92 ± 0.84	2.99 ± 0.32	3.46 ± 0.30	4.26 ± 0.67	0.004* (0.004)	< 0.001* (0.59)
Step duration (s)	2.3 ± 0.23	3.0 ± 0.22	3.80 ± 0.22	2.19 ± 0.19	2.88 ± 0.19	3.58 ± 0.23	< 0.001* (0.11)	< 0.001* (0.91)
Step size (°)	72.81 ± 8.50	62.72 ± 9.04	59.13 ± 8.07	79.69 ± 9.92	67.69 ± 8.73	62.52 ± 8.99	0.002* (0.08)	< 0.001* (0.46)

^+^Significant interaction (*p* < 0.05) and *significant main effects (*p* < 0.05) from mixed model analysis of variance.

**Table 3:** Post hoc comparisons for the main effects of groups and turning speeds as shown by the mixed model analysis of variance, where no interactions were indicated.

Variables	Comparison	Mean diff (SE)	*p*-value	CI of diffs	
				Lower bound	Upper bound
Eye onset (s)	Slower vs faster	0.08 (0.02)	0.001*	− 0.017	0.088
	Slower to normal	0.04 (0.02)	0.295	− 0.135	−0.088
	Normal to faster	0.05 (0.02)	0.026*	0.004	0.090
	PD to control	0.14 (0.02)	0.406	− 0.019	0.046
Head onset (s)	Slower vs faster	0.13 (0.02)	< 0.001*	0.096	0.168
	Slower to normal	0.06 (0.01)	< 0.001*	0.023	0.090
	Normal to faster	0.08 (0.01)	< 0.001*	0.040	0.111
	PD to control	0.02 (0.01)	0.039	0.612	0.645
Thorax onset (s)	Slower vs faster	0.13 (0.02)	< 0.001*	0.098	0.169
	Slower to normal	0.06 (0.01)	< 0.001*	0.021	0.088
	Normal to faster	0.08 (0.01)	< 0.001*	0.044	0.114
	PD to control	0.02 (0.01)	0.062	− 0.001	0.045
Pelvis onset (s)	Slower vs faster	0.14 (0.02)	< 0.001*	0.099	0.172
	Slower to normal	0.06 (0.02)	< 0.001*	0.025	0.098
	Normal to faster	0.07 (0.01)	< 0.001*	0.039	0.109
	PD to control	0.02 (0.01)	0.135	− 0.006	0.042
Peak head yaw velocity (°s^−1^)	Slower vs faster	−135.14 (8.40)	< 0.001*	−155.911	−114.365
	Slower to normal	−48.75 (5.0)	< 0.001*	−61.09	−36.41
	Normal to faster	−86.39 (8.52)	< 0.001*	−107.43	−65.34
	PD to control	−15.59 (6.12)	0.013*	−27.787	−3.382
Peak head-thorax angular separation (°)	Slower vs faster	−7.77 (1.60)	< 0.001*	−11.71	−3.835
	Slower to normal	−1.77 (1.43)	0.662	−5.288	1.745
	Normal to faster	−6.00 (1.80)	0.004*	−10.403	−1.600
	PD to control	−3.59 (1.32)	0.008*	−6.206	−0.967
Peak head-pelvis angular separation (°)	Slower vs faster	−2.30 (0.81)	0.019 *	−4.299	−0.302
	Slower to normal	−1.22 (1.17)	0.901	−4.299	1.648
	Normal to faster	−1.08 (1.05)	0.927	−3.680	1.519
	PD to control	−3.87 (0.84)	< 0.001*	−5.525	−2.207
Number of steps (n)	Slower vs faster	1.14 (0.15)	< 0.001*	0.778	1.495
	Slower to normal	0.67 (0.14)	< 0.001*	0.316	1.021
	Normal to faster	0.47 (0.08)	< 0.001*	0.269	0.666
	PD to control	0.58 (0.10)	< 0.001*	0.974	0.786
Step frequency (Hz)	Slower vs faster	1.28 (0.13)	< 0.001*	0.957	1.608
	Slower to normal	0.83 (0.13)	< 0.001*	0.502	1.150
	Normal to faster	0.46 (0.05)	< 0.001*	0.275	0.638
	PD to control	0.05 (0.09)	0.569	−0.134	0.242
Step duration (s)	Slower vs faster	1.45 (0.05)	< 0.001*	1.315	1.574
	Slower to normal	0.75 (0.05)	< 0.001*	0.638	0.867
	Normal to faster	0.69 (0.05)	< 0.001*	0.574	0.811
	PD to control	0.15 (0.04)	< 0.001*	0.066	0.226
Step size (°)	Slower vs faster	−15.43 (1.99)	< 0.001*	−20.292	−10.562
	Slower to normal	−4.38 (1.95)	0.081	−9.148	0.380
	Normal to faster	−11.04 (2.03)	< 0.001 *	−16.007	−6.078
	PD to control	−5.08 (1.62)	0.002 *	−8.294	−1.866

*Diff* difference, *CI* confidence intervals, *PD *Parkinson’s disease.

*Significant difference (*p* < 0.05) from mixed model analysis of variance.

**Figure 2:**
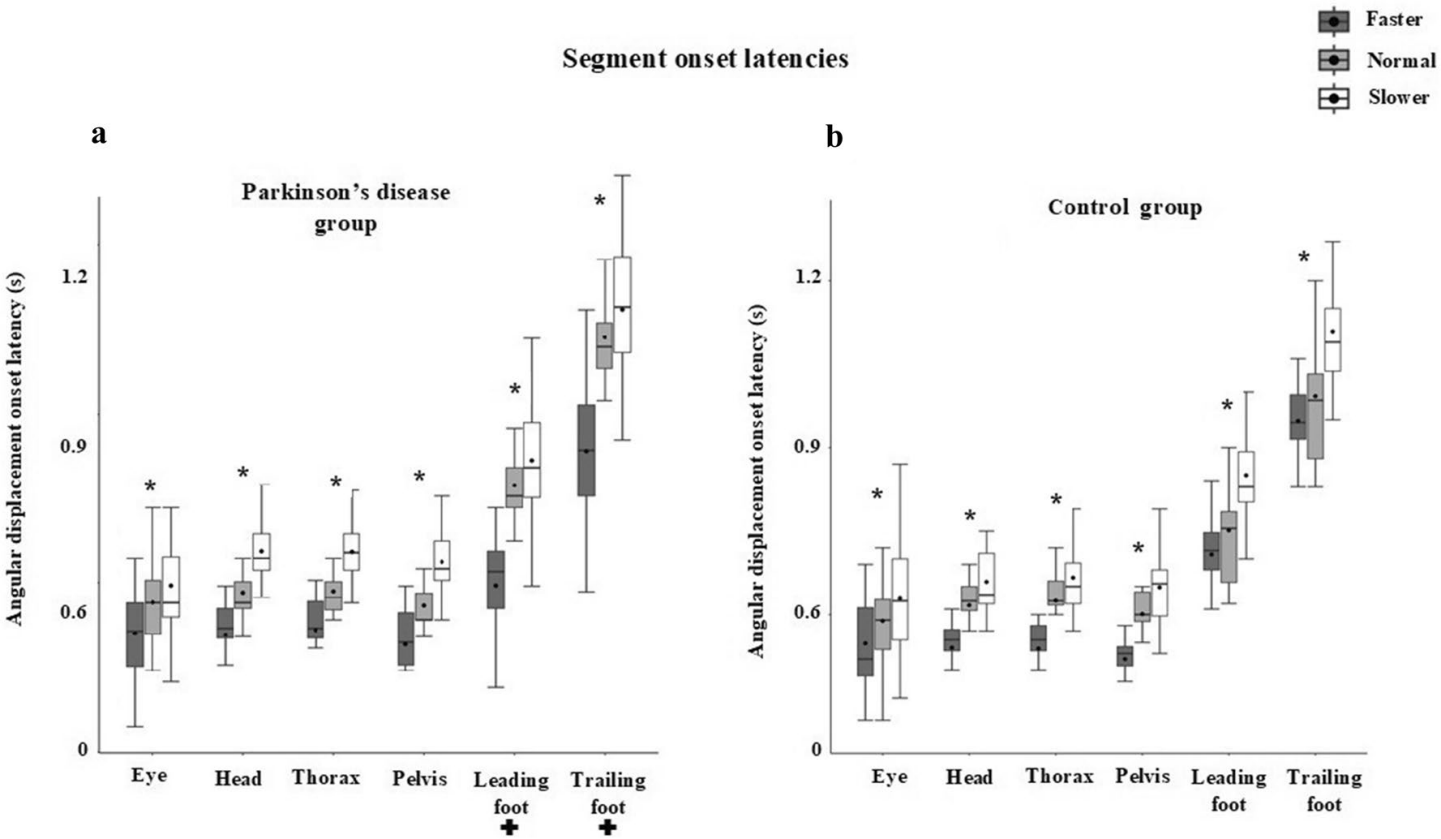
Boxplot demonstrating the mean angular displacement onset latencies of all segments with turning speed. Each segment onset latency was calculated with respect to the audio signal that cued the participant to initiate turning. (* shows significant main effect of turn speed and + shows significant interaction from the MM ANOVA).

The results from the turning kinematics and stepping variables are shown in Table 2. The MM ANOVA revealed significant interactions (*p* < 0.05) between groups and turning speed for the mean onset of latency for the leading foot (F = 6.00, η_p_^2^ = 0.14) and the trailing foot (F = 5.12, p = 0.008, η_p_^2^ = 0.12). A further analysis using RM ANOVA tests revealed a main effect of turning speed (*p* < 0.05) in the PD group and the post-hoc pairwise comparisons showed a significant increase between slower and faster speeds and normal and slower speeds. In addition, a significant main effect of turn speed (*p* < 0.05) was found on mean onset latency for all segments with onset latencies being longest during the slower selected speed and shortest during the faster selected speed which was seen in both groups (Fig. 2). A significant main effect between the groups was only found for head onset latency (*F*_(1, 19)_ = 6.57, *p* = 0.019, η_p_^2^ = 0.257), with the PD group demonstrating a longer head onset latency in comparison to the control group (Table 3). Surprisingly, segment reorientation onset latency showed similar characteristics in terms of simultaneous onset time for the eye, head, thorax, pelvis and feet, indicating the use of an en-bloc strategy by both groups (Fig. 2).

**Figure 3:**
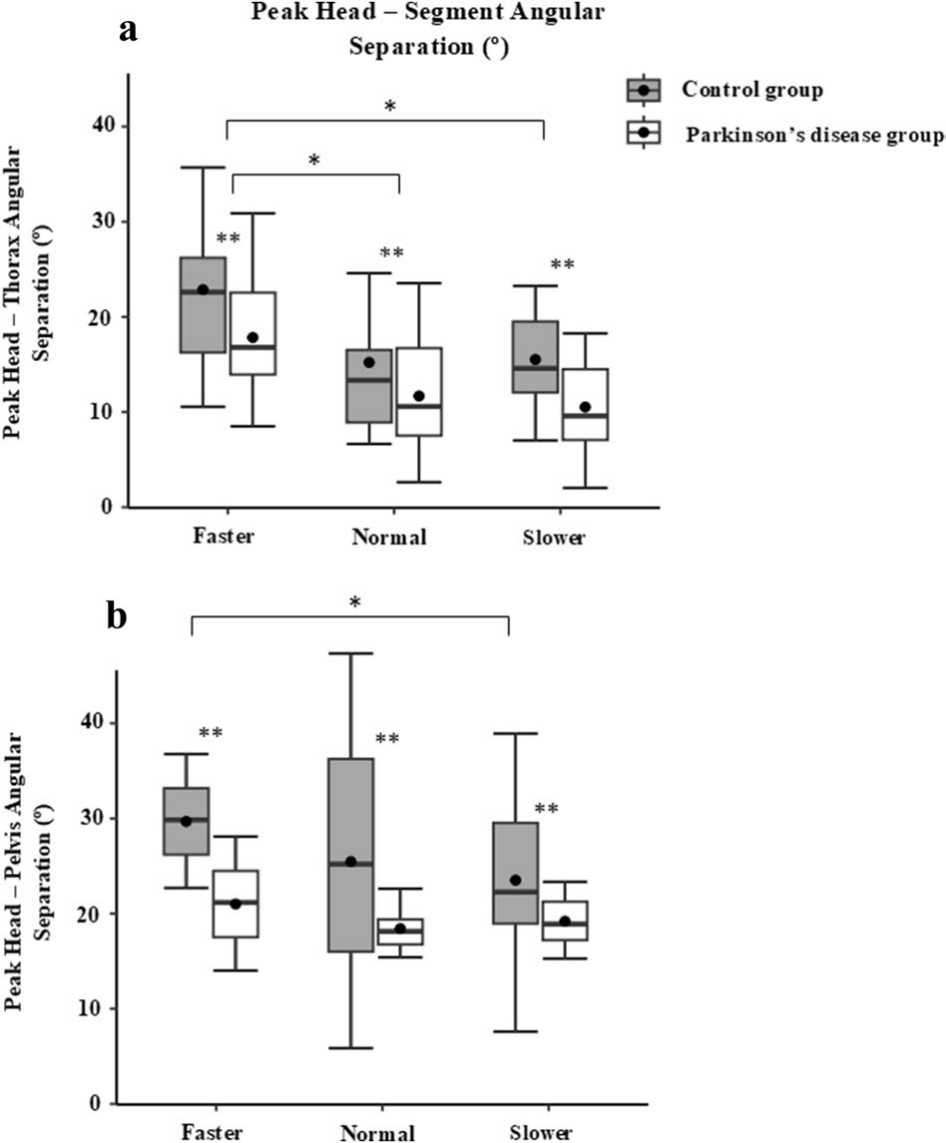
Boxplot demonstrates the median peak head-segment angular separation, with significant effects of turning speed on mean peak head segment angular separation in both groups for: (**a**) peak head-thorax angular separation and (**b**) peak head-pelvis angular separation. (*—significant main effect of turn speed and **—significant main effect of group).

For the intersegmental coordination, no significant interactions between groups and turn speed for peak head yaw velocity, peak head-thorax and peak head-pelvis angular separations were seen (Table 2). However, significant main effects were found between the groups (*p* < 0.05) and turning speed (*p* < 0.05) for all these variables (Table 1). Post-hoc pairwise comparisons revealed significant differences (*p* < 0.05) for peak head yaw velocity and also for peak head segmental angular separation between the faster and slower turning speeds, demonstrating that peak head yaw velocity and peak head-segmental angular separation increased with an increase in turning speed (Table 3). However, the PD group showed significantly smaller peak head-segmental angular separations than the control group (Fig. 3).

**Figure 4:**
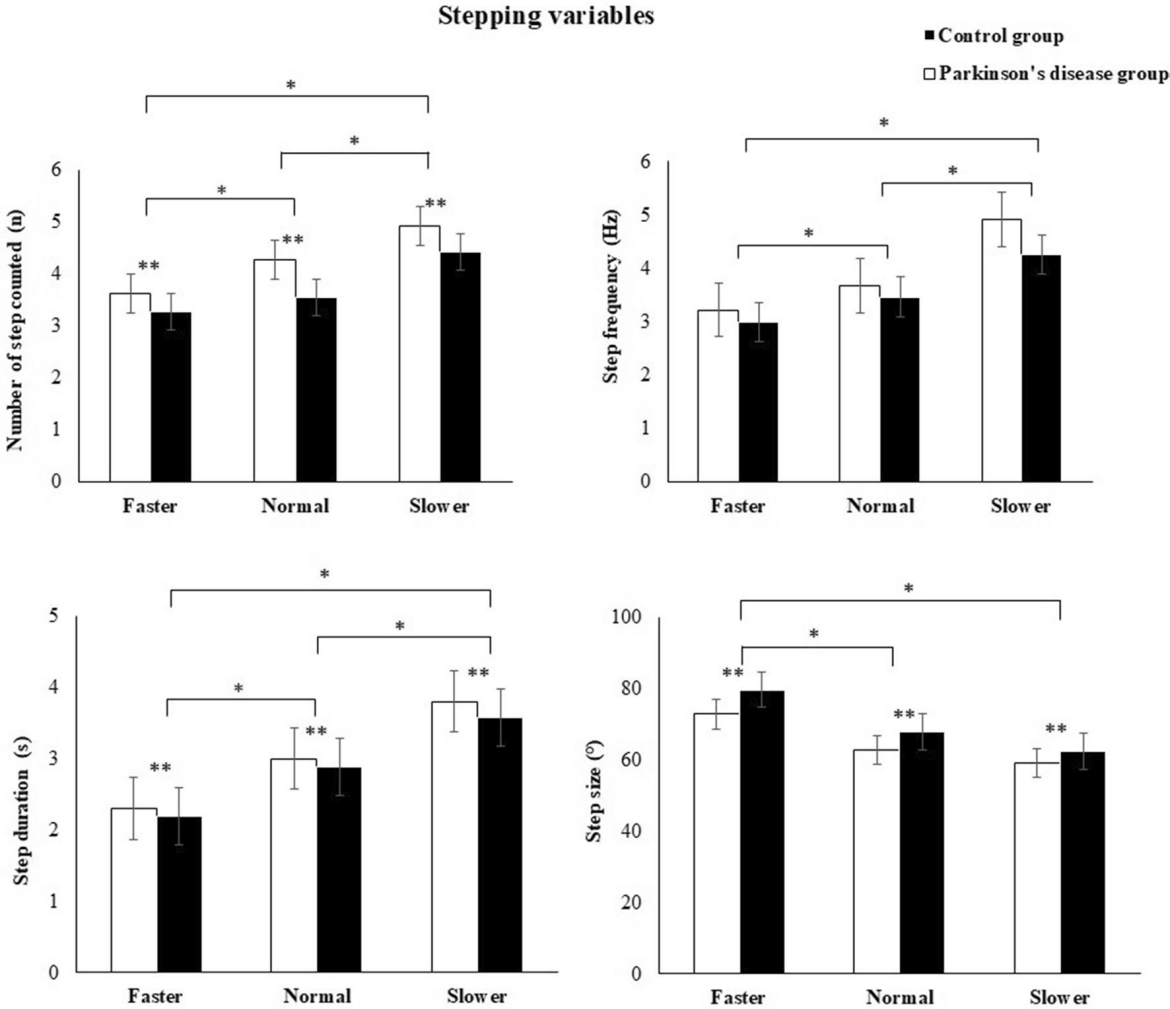
Bar graph of the results of **s**tepping variables showing changes in turning speed and differences between groups (*—significant main effect of turn speed and **—significant main effect of group).

The stepping variables (step count, step frequency, step duration and step size) between the two groups and three turning speeds are shown in Tables 2, 3 and Fig. 4. No significant interactions between groups and turning speed for stepping variables were found. However, the MM ANOVA revealed a significant main effect between the groups (*p* < 0.05) and turning speeds (*p* < 0.001). Further post-hoc pairwise comparisons showed the effects of turn speed were limited to all stepping variables, and significant differences were indicated between faster and normal speeds (*p* < 0.001), faster and slower speeds (*p* < 0.001) and slower and normal speeds (*p* < 0.001), which presented as the higher the step count, stepping frequency and step duration the smaller the step size made during slower turns than during faster turns. Also, the higher the step count, stepping frequency and step duration the smaller the step size while making normal speed turns than while making faster turns. In addition, a significant main effect (*p* < 0.05) was found between the two groups with people with PD showing a significantly increased number of steps counted, stepping frequency and step duration, and a decrease in step size when compared to the control group (Table 3, Fig. 4).

## Discussion

To the authors’ knowledge this is the first study to investigate the effects of turning speed during standing turns in individuals with and without PD. This study aimed to help our understanding surroundings the effects of changes in turning speed while turning in individuals with PD. Although group differences were observed in turning speed and turning characteristics, both groups showed the same response to turn speed with a clear top-down sequence of onset of body segment reorientation indicating that the relative timing between segments is preserved at each turn speed in both groups (Table 2). In other words, it seems that the relative timing sequence is the same for each turn speed but is initiated sooner for faster turns. These results may be explained by the methodology that the participants were cued to turn i.e. they initiated turning after watching an animation rather than responding to a cue light in their peripheral visual field or moving to a target beyond their field of view which they would need to visually identify^12,28,29^. Therefore, it is possible that visual information, and proprioceptive information from extraocular muscles about gaze direction, could be used to control the ongoing trajectory of the lower segments during turning and lead to the similar responses of turning characteristics between groups. In addition, turning slowly resulted in altered eye movement and turning kinematics and stepping characteristics in both the PD and the healthy group. However, this study did show that the speed of turning influences eye movement, turning kinematics and stepping behaviour, with a greater effect of speed of turning in the PD group when compared with the control group.

Significant interactions were seen between groups and turning speeds with regard to changes in eye movements including first fast phase amplitude and velocity characteristics, which were most notable in individuals with PD who turn significantly more slowly, made slower and smaller initial fast phase eye movements, and made more total fast phase eye movements than controls during standing turns. In addition, there was a main effect of turn speed on the number of fast phases, showing that the longer the turn duration the more eye movements they made. Eye movement and turning kinematics have been reported to be altered significantly in clinical populations with turning problems, and a causal link between oculomotor deficits and turning dysfunction has been suggested^12,28,30^. However, the effect of turn speed on the spatiotemporal fast phase characteristics in individuals without PD is unknown, therefore it is not possible to determine whether differences between groups are due to pathology caused by PD or simply alteration in behaviour by turn speed. In older adults and those with neurological conditions, turns frequently result in falling. The majority of research into turning has focused on these populations and studies have identified deficits in eye movements and problems with body segment reorientation, however the mechanisms resulting in falls in these individuals is still unclear. One possible explanation is that the fundamental oculomotor control system is preserved^29^. The movement of the eyes in conjunction with the head could be explained by the interaction between the vestibular and ocular systems that coordinate the movement of the two segments to ensure successful gaze transfer^12,15,28^. The oculomotor system has access to information from the visual cues and visual systems in obtaining spatial and egocentric information that is passed to the motor system^12,29^. The central nervous system (CNS) uses visual information to plan the postural adjustments and to coordinate whole-body responses^30–32^. Furthermore, the anticipatory eye and head movements is involved in the synergy of movement during turning^30,32,33^. Taking these together, it is likely that the motor symptom of PD such as bradykinesia and rigidity may limit head movement resulting in both anticipatory head and eye movement. This would lead to disruption of the spatial and egocentric information used in planning the motor response in individuals with PD in comparison to individuals without PD. Thus, the CNS provides the primary motor pattern that can be adopted to control similar motor tasks, reducing both the complexity of motor planning and the reliance on sensory feedback^30,32^. Several previous studies which focussed on eye movement and turning in individuals with PD have reported that individuals with PD exhibit a greater number of saccades during turning and show differences in initial fast phase amplitude and velocity when compared with healthy controls^12,27,29,33^. Our results show the same trends in eye movement characteristics in individuals with PD and the same differences between the groups. The earlier studies also suggested that it seems possible that some cardinal features in terms of bradykinesia and rigidity are responsible for the observed dysfunction of saccadic eye movement, which may be due to PD neuropathology^12,29^.

Individuals with PD demonstrated the same pattern of segment onset latencies compared to the healthy older adult group. Our results consistently support the findings of previous studies that have documented that when visually cued to turn, individuals with PD take longer to initiate axial segment rotation than neurotypical control participants which suggests that bradykinesia could account for these differences^3,8,12,28,30^. In addition, this current study confirms a top-down sequence of the onset of body segment reorientation in healthy adults during turning. The horizontal movement starts with the eyes, which shifts the gaze towards the new direction of travel; this is followed by head trunk yaw and reorientation of the feet. However, when considering the rotation initiation between groups, individuals with PD presented with a delay in reorientation onset for all segments when considering the onset latency of all segments, and it was found that the faster the turn the earlier the rotation onset of the sequence. These findings support a control synergy schema set of motor patterns that are part of a core motor program for human movement which may be extended to regulate comparable motor activities dependent on sensory feedback^27,28,30^. This suggests that segmental reorientation onset latency may be adopted to simplify control and may be an indicator of compensation to maintain balance and stability when completing standing turns at different speeds. These results show that the main differences previously identified between PD and age-matched controls are likely a consequence of neuropathology^12,17,27–30,34^. Previous studies also found that individuals with PD had longer onset latencies of body segments in response to a trigger to turn in association with a longer turn duration, which may be explained by the bradykinesia affected motor systems^28,35^. The slow turning in PD resulting from the bradykinesia has the consequence of causing difficulty in performing daily activities, especially with regard to turning or sequential movements and leads to the inability to start and stop movement, increasing the risk of falls. An adaptive strategy allows a more effective and slower control of movement, as proposed in the general theory of bradykinesia, and is considered to be a compensatory strategy to reduce the variability of the resulting motor performance^12^. Our results suggest that one of the mechanisms of turning dysfunction is a by-product of slow turning speed due to pathologically induced bradykinesia in people with PD.

It is important to note that significant differences were found in the peak head yaw velocity and angular segment separations and turn speed for both groups. In the PD group, with the peak head velocity being significantly lower in comparison to the control group. This may be as a result of the disruption of head-in-space rotation effect which could interfere with the goal of moving the head in the direction of the turn and may be related to the en-bloc findings in the PD group, as this strategy was observed more frequently than in the healthy control group^12,36,37^. These findings support the rationale that head movement commands the signal and coordinates during predictable conditions as a top-down mechanism between body segments^12,28^. Similar to a previous study, it was found that individuals with PD and age matched controls have reduced head on trunk rotation with a more en-bloc strategy turning the body segments simultaneously during turning. In addition, Anastasopoulos and co-wokers in 2011 indicated that a slowness of trunk and a lesser degree of head on trunk rotation resulted from insufficient muscle recruitment and bradykinesia in PD^12^. One explanation described in the previous study suggested that an en-bloc pattern of segmental reorientation during turning may be adopted to simplify the control of turning, and may be an indicator of compensations for decreased stability and balance; therefore, it may be useful in identifying older adults at risk of falling^3,25,28^. Additionally, a decrease in the degree of separation between the head and the trunk segments with concurrent changes in turning characteristics may help in providing the eyes with the most appropriate head-on-body position in order to gain advance visual information. Another possible relevance of decreasing head-on-body movement is turn performance which may help to elucidate the decreases in whole-body coordination. Interestingly with regard to the second point, our results demonstrated that in the PD group alteration of the main sequences of fast phase characteristics in terms of fast phase amplitude and velocity, were greater than the healthy controls. This could explain that the gaze control system is contained to ensure that the gaze consistently triggers the direction of the turn in advance of the head and body rotation. This suggests that either visual information about environmental features or proprioceptive information from the extraocular muscles about gaze direction could be used to control the ongoing trajectory of the lower segments.

This study found that the foot rotation during the swing phase (step size) reduced and the number of steps, step frequency and step duration increased during slower turns. Also, in the PD group, the crucial step variables identified were a narrower step, and a higher step frequency and step duration in comparison to the control group. Our results were consistent with the findings of previous studies which reported on individuals with PD in comparison to healthy older adults^3,19,27–29^. One rationale to explain these results is that the reduction in head-on-trunk rotation in people with PD in comparison to controls affects the stepping processes that are responsible for the maintenance of balance during turning. In addition, our findings support the concept of the stepping motor control system. The step placement during pre-planned turns could be part of a planned mechanism, and central commands for rotational movements of the lower extremities during turning may utilise neuronal networks in the spinal cord sub serving locomotion^7,19,38^. Hence, the step actions during turning are critical as they primarily require the control of the centre of mass by changing the width of base of support or mechanical muscle demand, which can directly influence the strategies employed to maintain stability and balance. Individuals with PD have poor gait stability caused by disruption of the centre of mass and centre of gravity inclination angle, which results in individuals with PD taking extra turning time and turning steps to increase stability^19^. In people with PD who had an abnormal gait, an attempt was made to increase their step width to maximise the base of support and resulted in changing in stepping characteristics during turning^37^. Shuffling gait results in an involuntary forward trunk lean, leading to a constant state of postural instability and reduces the medial deviation with a forward shift of the centre of mass. In combination with our results, these findings suggest that stepping characteristics in people with PD during turning may be an indirect result of perception of the speed of turning or actual instability.

Previous research also suggests that the taking of many small steps may result in freezing episodes and is a debilitating common characteristic of gait in individuals with PD leading to difficulties during initiating walking, turning, changing direction, changes in the environment and also cognitive changes^5,39^. It has also been shown that individuals with PD who exhibit freezing of gait show a reduction in medial deviation and a forward shift of the centre of mass (COM)^39^. The freezing of gait may occur in order to preserve postural stability and maintain COM in a wider base of support throughout the turn. In addition, the difficulties in changing segment orientation and altered top-down coordination may all contribute to problems in postural control during turning^5^. Therefore, it is likely that freezing gait is one of the factors that can put individuals with PD at a greater risk of losing balance and falling while turning.

Turning while standing and stepping require effective strategies to correct balance and prevent falls^25,40^. Consequently, a forward stepping strategy has been used to initiate and reduce the amplitude of the step during turning^37^. This is supported by King and colleagues in 2012 who suggested that the severity of the stage of PD, especially in those who have prominent bradykinesia and obvious equilibrium impairment, and even in individuals with mild PD who do not have gait abnormalities, had impact on turning ability during standing turns or walking^41^.

There are several limitations to this study. Firstly, we recruited individuals with PD in modified Hoehn and Yahr stages 2.5–3 who were able to walk independently. The findings in this study may not be applicable to individuals with PD who are at other modified Hoehn and Yahr stages. Secondly, the findings of this study indicate that further research be conducted on the assessment of turning with the inclusion of the freezing of gait, which is related to locomotion in PD. Furthermore, to learn more about how turning deficits change in PD, the kinetics assessment and dual tasks of standing turns should be prioritized, and walking turns in individuals with PD should be investigated. Thirdly, the investigation into turning in this study featured individuals with PD in an "on" medication state; further study is needed to examine and compare this with their "off" state. Future studies need to focus on the clarification of distinction between the stages of turns, which occur between starting from a static posture and continue to ongoing locomotion.

When beginning a turn from a quiet stance, anticipatory postural adjustments, biomechanics and sensory mechanisms are likely to be different from those which occur during walking. Further studies could also investigate other factors involved during the exercise programme, such as eye movement training, behavioural modification, sensory integrations training or cognitive training, which may be more effective and more advantageous for individuals with PD. Assessment of the long-term effects of exercise on turning performance and a study using a greater number of participants is necessary.

## Conclusion

The speed of turning has an effect on the following dependent measures in both individuals with PD and age matched controls: onset latency of the eye, head, thorax, and feet; peak head-segment angular separation; and step variables, but these occurred in a more pronounced manner in individuals with PD in response to turn speed. Slow speed of turning results in an increase in the degree of difficulty when performing activities of daily living in people with PD, especially with regard to turning or sequential movements leading to the inability to start and stop movement, which increases the risk of falls. These findings indicate that the speed of turning is an important factor to take into account during the assessment of standing turns in individuals with PD and improves our understanding of the strategies adapted in challenging situations. Therefore, these findings help our understanding regarding the turning strategies in individuals with PD and may have implications for physical therapists and would support adding speed of turning training to help to improve or limit the problems associated with turning difficulties.

## Data Availability

The data presented in this study are available from the corresponding author upon reasonable request.
